# Sunscreen on Screen: Cross‐Sectional Study of TikTok Content on Sunscreen

**DOI:** 10.1111/srt.70061

**Published:** 2024-10-04

**Authors:** Shahrin Khan, Moaid Shaik, Nawrin Khan, Geoffrey Potts

**Affiliations:** ^1^ School of Medicine Wayne State University Detroit Michigan USA; ^2^ College of Osteopathic Medicine Michigan State University East Lansing Michigan USA; ^3^ Department of Dermatology Wayne State University Detroit Michigan USA

Dear Editor,

TikTok is an increasingly popular social media platform where users can share and view videos often shorter than 3 min. The application has a large, young audience that makes up its user base [1].

In the context of dermatology, the application is a space where health information can be shared and received. Keeping in mind topics such as treatment of acne vulgaris, various skin concerns, and prevention of sun damage, it is important to understand the quality of the knowledge being disseminated across this application. The number of likes and view count determine the popularity of posts, but it may be inversely proportional to the actual quality of the information being shared [2]. Another study investigated the strong presence of misinformation as it relates to psoriasis content [3]. This study assesses the quality of TikTok videos regarding sun protection.

Under the search term “#sunscreen” with over seven billion views, the first 100 of the most popular videos were chosen on April 8, 2023 using the inclusion criteria of relating to sunscreen and being in English. Descriptive information was subsequently collected regarding each video such as the type of content creator (and if they are a board‐certified physician), their gender, date of upload, and video category. The effectiveness and quality of content portrayed in each video were assessed using the DISCERN instrument. This reliable 16‐question tool allows for the appraisal of consumer health information on treatment choices using a 1 (very poor) to 5 (excellent) scale [4]. Two reviewers assessed the 100 videos, and Cohen's *κ* coefficient was then calculated for inter‐rater reliability.

Table 1 provides a comprehensive overview of the qualities of the 100 most popular videos on TikTok regarding sunscreen. An overall mean DISCERN score was 2.68. Cohen's *κ* coefficient demonstrated high inter‐rater reliability at *κ* > 0.8. Among the 100 videos, 12 were created by board‐certified physicians, who had a higher average DISCERN score of 2.83 than non‐physicians at 2.68. Across all TikTok videos, shortcomings included not providing reference to sources, descriptions of the mechanism of sunscreen, or support for shared decision‐making. Many videos centered around different brands of sunscreen and user reviews of their appearance on the skin. This includes its presentation on people of color, who may experience a “white cast” with various products. The primary risk of not regularly using sunscreen highlighted in many videos is aging over skin cancer and pigmentary disorders.

**TABLE 1:** Qualities of the top 100 popular TikTok videos on sunscreen.

	Number	Mean DISCERN score
Type of creator		
Physician	12	2.83
Non‐physician individual	87	2.70
Private company	1	1.00
Gender		
Male	17	2.76
Female	82	2.68
Other	1	1.00
Video categories		
Personal anecdote	37	2.19
Review of sunscreen product(s)	30	3.10
Educational information on sunscreen	28	3.11
Advertisement	5	1.40

Overall, TikTok videos are rated as having significant shortcomings with regard to health education on sun protection. Therefore, per DISCERN, we suggest providing evidence‐based sources on created content to improve reliability. Content made by physicians can have higher share counts when compared to other creators [5]. Patients can build interactive relationships with dermatologists on these platforms to be educated on the benefits of sunscreen and a routine that prevents skin damage. As the online presence of dermatologists continues to grow, the quality of health information available to users can and should be improved.

## Ethics Statement

No human or animal subjects were involved in this study.

## Conflicts of Interest

The authors declare no conflicts of interest.

## Data Availability

The data that support the findings of this study are available from the corresponding author upon reasonable request.
